# Measurement of the area of venous ulcers using two software programs**^1^**

**DOI:** 10.1590/1518-8345.1673.2862

**Published:** 2016-12-19

**Authors:** Thaís Dresch Eberhardt, Suzinara Beatriz Soares de Lima, Luis Felipe Dias Lopes, Eline de Lima Borges, Teresinha Heck Weiller, Graziele Gorete Portella da Fonseca

**Affiliations:** 2 Doctoral student, Departamento de Enfermagem, Universidade Federal de Santa Maria, Santa Maria, RS, Brazil.; 3 PhD, Adjunct Professor, Departamento de Enfermagem, Universidade Federal de Santa Maria, Santa Maria, RS, Brazil.; 4 PhD, Associate Professor, Departamento de Administração, Universidade Federal de Santa Maria, Santa Maria, RS, Brazil.; 5 PhD, Associate Professor, Departamento de Enfermagem, Universidade Federal de Minas Gerais, Belo Horizonte, MG, Brazil.; 6 Master's student, Departamento de Enfermagem, Universidade Federal de Santa Maria, Santa Maria, RS, Brazil.

**Keywords:** Nursing, Varicose Ulcer, Weights and Measures, Software Validation

## Abstract

**Objective:**

to compare the measurement area of venous ulcers using AutoCAD^(r)^ and Image Tool software.

**Method:**

this was an assessment of reproducibility tests conducted in a angiology clinic of a university hospital. Data were collected from 21 patients with venous ulcers, in the period from March to July of 2015, using a collection form and photograph of wounds. Five nurses (evaluators) of the hospital skin wound study group participated. The wounds were measured using both software programs. Data were analyzed using intraclass correlation coefficient, concordance correlation coefficient and Bland-Altman analysis. The study met the ethical aspects in accordance with current legislation.

**Results:**

the size of ulcers varied widely, however, without significant difference between the measurements; an excellent intraclass and concordance correlation was found between both software programs, which seem to be more accurate when measuring a wound area >10 cm².

**Conclusion:**

the use of both software programs is appropriate for measurement of venous ulcers, appearing to be more accurate when used to measure a wound area > 10 cm².

## Introduction

Venous ulcers are among the chronic conditions that affect the population and require differentiated management of nursing care. The prevalence is estimated at 0.5 to 0.8%, with an incidence between two and five new cases per thousand per year^1^.

Venous ulcers significantly affect the quality of life of individuals, with repercussions at work, in social relationships, and limitations in their daily routines^2^. Furthermore, they cost 900-1000 EUR when requiring three to six months for healing^1^. 

Considering this scenario, the nurse has an important role in the evaluation of these patients^2^ ^-^ ^3^ and should use available technologies to conduct this process. Thus, the measurement is an objective way to evaluate the wound and identify the progress of healing.

In addition to the importance of the subject being studied, a gap remains in the knowledge produced^4^. Among the software available for measurement, two programs can be used: AutoCAD^(r)^ software and the Image Tool. The first is a program commonly used by engineers in topography for physical area calculation^5^ and the second was developed by the University of Texas Health Sciences Center at San Antonio, and is a free software used as a tool to obtain objective and reliable measures to the real size of the lesion^6^.

Based on the above, the objective was to compare the measurement of the area of venous ulcers using AutoCAD^(r)^ and Image Tool software.

## Method 

This was a reproducibility study of evaluation tests. The study was conducted in a angiology clinic of a university hospital in southern Brazil, which treats patients with venous ulcers. The patients were selected according to the following inclusion criteria: age over 18 years, intact cognitive and verbal skills, presenting venous ulcers covering one side of the lower limb. 

Five nurses who were participants in the skin wound study group of the hospital where the research was conducted, called evaluators, were responsible for data collection, which occurred from March to July of 2015. All nurses were trained in the process of obtaining the photographs and wound measurement.

In the collection period, among 48 patients with venous ulcers, 21 met the inclusion criteria, totaling 36 venous ulcers, 72 photographs and 144 measurements in each software program. The researcher and one of the evaluators, who was present at the time of ulcer dressing change, photographed the venous ulcers. Both photographs were taken during the same care period. Next, the information was transferred to the researcher's notebook. The evaluator and the researcher performed the measurements of both photographs. This process was performed with all evaluators, always in pairs (researcher and one evaluator), until reaching the maximum number of individuals of the study population.

The data collection procedure occurred according to the following protocols. 

-Protocol for obtaining photographs of wounds: after cleaning the wound with 0.9% saline solution, a surgical compress was placed under the lower limb with the ulcer, in order to give a white background color for the picture; a black square with three centimeters printed in on A4 sheet was placed close to the ulcer, to be considered as a reference object. The photograph was taken with a Fujifilm Camera FinePix S14 megapixels (f/6.4, ISO 400, enabled macro function, automatic white balance, high sharpness, and flash disabled). The camera was perpendicular to the wound (90°), and 50 cm away from the wound, bringing it closer to or farther away from the wound, as necessary.

-Protocol for wound area measurement using the AutoCAD^(r)^ software (software 1): based on the manual from the Federal University of Santa Catarina^7^. The 2015 version was used, with educational license for student registration Nº 900-5013697. To obtain the area of a venous ulcer, in cm², the following formula was used:

-Protocol for wound area measurement using the software Image tool (software 2): based on the information described in the article, *Software Image Tool 3.0 an instrument for measuring the wounds*, published in 2012^6^.

Data were uploaded into Microsoft Office Excel^(r)^, using duplicate, independent entry in order to correct any typographical errors. For the statistical treatment of the area of the venous ulcers, mean and standard error were used. In order to identify the normality of the data distribution, the Shapiro-Wilk test was applied; due to lack of data normality, the Wilcoxon test was used in order to identify differences between the measurements, and to verify the degree of systematic differences between measurements in pairs (researcher and evaluator).

The distribution of the differences between the measures and the mean difference between them was analyzed^8^. The reproducibility was measured using the intraclass correlation coefficient (ICC) and concordance correlation coefficient (CCC) of Lin^8^. The correlation was considered low for values < 0.40, moderate for values between 0.40 and 0.75, and excellent for values > 0.75.

The Bland and Altman procedure^9^ was performed only on normally distributed data, after performing the logarithmic transformation. For analysis purposes, a significance level of 5% and confidence interval (CI) of 95% were used Analyses were performed using the R statistical program.

The ethical principles were based on the National Council of Health Resolution No. 466 of December 12, 2012. The project was submitted to the Research Ethics Committee and was approved, protocol No. 932838 and (CAAE) No. 40250814.6.0000.5346. The research was conducted after the Terms of Free and Informed Consent Form was signed by participants.

## Results

**Figure 1:**
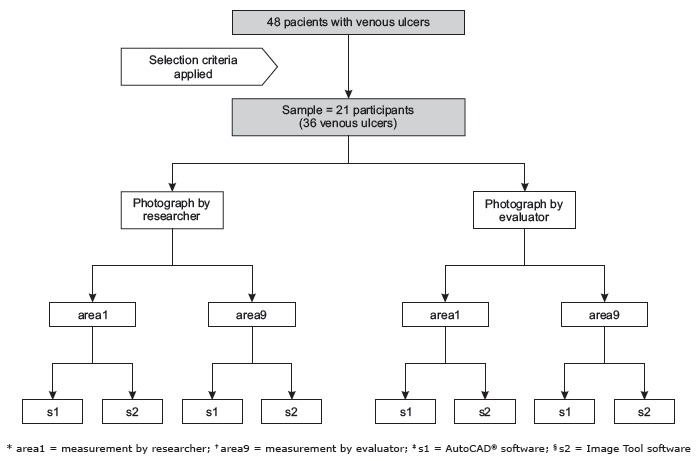
Diagram of the study participants selection and data collection procedure, Santa Maria, RS, Brazil, 2015

The study participants (n = 21) had a mean age of 60.9 years, and nine (42.9%) were aged 64-72 years; the majority were male (66.7%). The number of venous ulcers ranged from one to five per patient, with a median of 1.8, totaling 36 venous ulcers (Figure 1).

**Figure 2:**
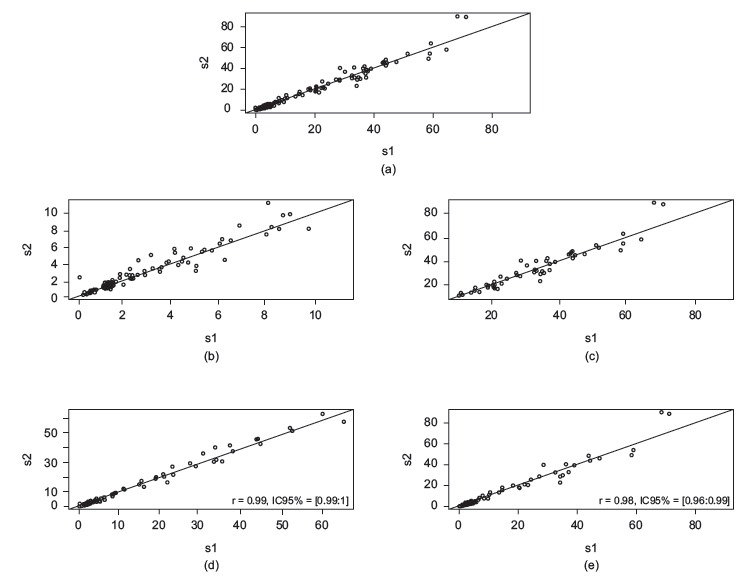
a) Scatterplot of measured areas in AutoCAD^(r)^ (s1) and Image Tool (s2) software; b) Scatter plot of the areas ≤ 10 cm² measured in AutoCAD^(r)^ (s1) and Image Tool (s2); c) Chart <dispersion of areas> 10 cm² measured in AutoCAD^(r)^ (s1) and Image Tool (s2); d) Scatter plot of the areas measured by the researcher in AutoCAD^(r)^ (s1) and Image Tool (s2); e) Scatter plot of the areas measured by the evaluators in AutoCAD^(r)^ (s1) and Image Tool (s2), Santa Maria, RS, Brazil, in 2015.

The areas measured by software 1 ranged from 0.2 to 71.0 cm², with a mean of 14.4 ± 1.4. The areas measured by software 2 ranged from 0.4 to 89.1, with a mean of 14.9 ± 1.5. The dispersion of the measures are demonstrated in Figure 2, and the mean observed difference of 1.6 ± 0.2. No difference (p = 0.80) was identified between the measurements performed by the two software programs. There were excellent ICC [ρ = 0.98; 95% (0.98-0.99); p < 0.05] and CCC [0.95; IC 95% (0.92-0.97) values.

No difference was found between the measurements of venous ulcers ≤ 10 cm² (p=0.64) with the two software programs, with an area > 10 cm² (p=0.92) between the measurements of the researcher (p=0.80) and evaluators (p = 0.90).

The ICC and CCC values were excellent for all comparisons, as shown in Table 1.

**Table 1:** Intraclass correlation coefficient and concordance correlation coefficient of venous ulcers measured by AutoCAD^(r)^ and Image Tool software. Santa Maria, RS, Brazil, 2015

Measurements	ICC* (CI^†^ 95%)	p-value	CCC^‡^(CI^††^ 95%)
The measurements	0.98 (0.98-0.99)	< 0.05	0.95 (0.92-0.97)
Area ≤ 10 cm²	0.96 (0.93-0.97)	< 0.05	0.95 (0.93-0.97)
Area > 10 cm²	0.96 (0.92-0.97)	< 0.05	0.95 (0.92-0.97)
Measurement by researcher	0.99 (0.99-0.99)	< 0.05	0.99 (0.99-0.99)
Measurement by evaluator	0.98 (0.96-0.99)	< 0.05	0.97 (0.96-0.98)

* ICC = Intraclass correlation coefficient. ^†^ IC = Confidence interval. ^‡^ p-value of intraclass correlation

**Figure 3:**
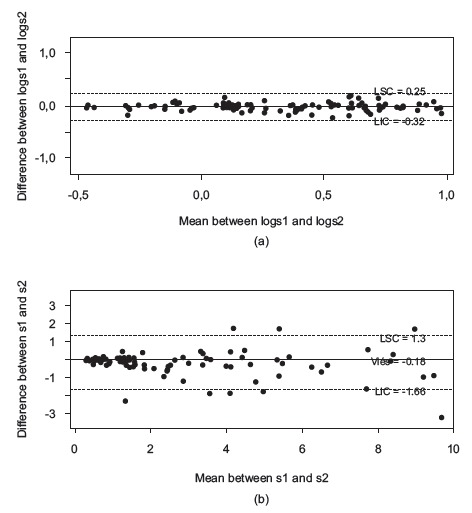
a) Bland-Altman plot for the difference between means and areas >10 cm², measured in AutoCAD^(r)^ (s1) and Image Tool (s2); b) Bland- Altman plot for the difference between means and areas ≤10 cm², measured in AutoCAD^(r)^ (s1) and Image Tool (s2), Santa Maria, RS, Brazil, 2015

The Bland-Altman plot (Figure 3) shows the correlation between the measurements in both software programs. This analysis was performed only for the two categories that showed normal distribution after log transformation (ulcers ≤ 10 cm² and> 10 cm²).

For ulcers with an area >10 cm², the upper concordance limit (UCL) was 1.26 cm², and the lower concordance limit (LCL) was 0.74 cm², with a measure outside these limits. At this time, for the wounds with an area ≤10 cm², a UCL of 1.8 cm² was found and a LCL of 0.02 cm², and a variety of measures outside these limits were displayed.

## Discussion

The sizes of ulcers varied widely; however, there was no statistically significant difference between the measurements. Other studies that characterized patients with venous ulcers in outpatient care also found wide range of wound sizes^10^ ^-^ ^11^, corroborating the findings of this research. Still, it may indicate that venous ulcers are wounds that have different sizes.

The ICC and the CCC data demonstrate that the measurements have intraclass correlation and excellent concordance, i.e., the use of both software programs is suitable for the measurement of venous ulcers.

However, both software programs seem to be more accurate when used to measure large wounds (with an area > 10 cm²), as the limits of agreement were clinically acceptable, and only one measurement was out of bounds. Considering that, when analyzing small wounds (area ≤ 10 cm²), the UCL was clinically questionable and there were several measurements outside the limits of concordance.

Another study^5^ that compared the AutoCAD^(r)^ software with another program also found that marking offsets, the relative point of view, is larger in wounds with a smaller area, since it requires hand movements of the operator.

One study^12^ compared three methods of pressure ulcer area measurement - ruler, tracing paper with graduated acetate, and digital planimetry; it found that all three methods were appropriate for measuring the surface area of small circular wounds (area ≤ 10 cm²); however, in irregularly shaped wounds > 10 cm², the ruler overestimated the size.

A systematic review^13^ that evaluated the performance of instruments designed to measure the dimensions of pressure ulcers found that digital photography, combined with software for the measurement of wounds, demonstrated satisfactory agreement.

The limitations of this study are: the type of sampling (non-probabilistic), the lack of studies available on the subject, and the lack of training on basic concepts regarding the evaluation of wounds, such as defining the surface and wound edges.

Further studies are suggested with this software, with comparisons between invasive and non-invasive methods, and analyzing different types of chronic wounds. Still, the incorporation of digital photographs and measurement by means of computer programs is supported, as it enables a most accurate record of the aspects and measures of the wound.

## Conclusion

The sizes of the ulcers showed great variance, however no statistically significant difference was found between the measurements made with the two software programs. The intraclass correlation coefficient and agreement were excellent, and both software programs were suitable for the measurement of venous ulcers; they may be more accurate when used to measure wound areas > 10 cm².
